# The Role of Left Supplementary Motor Area in Grip Force Scaling

**DOI:** 10.1371/journal.pone.0083812

**Published:** 2013-12-31

**Authors:** Olivier White, Marco Davare, Michaël Andres, Etienne Olivier

**Affiliations:** 1 Institute of Neuroscience, Université catholique de Louvain, Brussels, Belgium; 2 Unité de Formation et de Recherche en Sciences et Techniques des Activités Physiques et Sportives, Université de Bourgogne, Dijon, France; 3 Institut National de la Santé et de la Recherche Médicale, Unité 1093, Cognition, Action, and Sensorimotor Plasticity, Dijon, France; 4 Sobell Department of Motor Neuroscience and Movement Disorders, UCL Institute of Neurology, University College London, London, United Kingdom; 5 Institut de recherche en sciences psychologiques, Université catholique de Louvain, Louvain-la-Neuve, Belgium; Katholieke Universiteit Leuven, Belgium

## Abstract

Skilled tool use and object manipulation critically relies on the ability to scale anticipatorily the grip force (GF) in relation to object dynamics. This predictive behaviour entails that the nervous system is able to store, and then select, the appropriate internal representation of common object dynamics, allowing GF to be applied in parallel with the arm motor commands. Although psychophysical studies have provided strong evidence supporting the existence of internal representations of object dynamics, known as “internal models”, their neural correlates are still debated. Because functional neuroimaging studies have repeatedly designated the supplementary motor area (SMA) as a possible candidate involved in internal model implementation, we used repetitive transcranial magnetic stimulation (rTMS) to interfere with the normal functioning of left or right SMA in healthy participants performing a grip-lift task with either hand. TMS applied over the left, but not right, SMA yielded an increase in both GF and GF rate, irrespective of the hand used to perform the task, and only when TMS was delivered 130–180 ms before the fingers contacted the object. We also found that both left and right SMA rTMS led to a decrease in preload phase durations for contralateral hand movements. The present study suggests that left SMA is a crucial node in the network processing the internal representation of object dynamics although further experiments are required to rule out that TMS does not affect the GF gain. The present finding also further substantiates the left hemisphere dominance in scaling GF.

## Introduction

One remarkable feature of human beings is their exquisite dexterity, leading to an unrivalled ability to use tools. This capacity of utilizing common objects and tools appropriately as soon as we grasp them relies, amongst others, on the ability to determine in advance the grip force (GF) required to handle them in relation to their mechanical properties and the surrounding environment. This anticipatory strategy permits to apply the appropriate GF as soon as the fingertips contact the object, avoiding the uncompressible delays in the sensorimotor system [1]. The predictive control of GF is made possible because the nervous system can learn, store and then select the internal representations of the dynamics of innumerable objects, known as “internal models” [2], [3]. The anticipatory control of GF has been studied in great detail because it has been regarded as evidence for the existence of forward models in the brain [4], [2], [5], [6], [7].

Although the implementation of internal models of object dynamics in the nervous system is widely acknowledged, their neural correlates are still discussed. While many neurological conditions alter GF scaling, clinical studies have not been very informative in identifying the neural correlates of internal models, mainly because deficits in GF scaling reported in patients are usually too crude to determine the precise role of a given cortical or sub-cortical structure in its control [8], [9], [10]. Functional neuroimaging studies using a standard grip-lift task [11], [12] also failed to identify the neural correlates of internal models because this task typically leads to the activation of a large number of cortical areas [13], [14], [15], probably responsible for encoding concomitantly other movement parameters. Only more recently, the use of innovative experimental paradigms, combined with functional imaging techniques, has permitted to identify brain regions specifically involved in the implementation of internal models [16]. There is a large consensus in the literature that the cerebellum plays a key role in acquiring and storing internal models [16], [5], [17], [18], [19] and, in particular, in coupling the GF and load forces (LF) [20]. The primary motor cortex (M1) has also been regarded as an important node in the network responsible for learning internal models [21], [16] although it has been suggested that M1 activation may largely reflect the adaptation of muscle activity correlated with changes in dynamics [22].

Another cortical area frequently considered as a possible candidate for implementing internal models is the supplementary motor area (SMA). Indeed, Imamizu and colleagues have reported an increase in SMA activation after learning new internal models [23] and Bursztyn and colleagues suggested that SMA could be responsible for loading the appropriate internal model, once selected [16]. In order to gain further insight into the role of SMA in controlling GF, we used transcranial magnetic stimulation (TMS) to interfere with the normal functioning of this area while healthy volunteers were involved in a standard grip-lift task with either hand. By performing transient virtual lesions, TMS allows us to investigate the causal role of this area in the task at hand, with a good time resolution.

## Materials and Methods

### Subjects

The present study consists in two SMA experiments performed on 14 healthy volunteers (10 males, aged 28±4.2 years, mean ± SD) and a third control experiment, involving five additional participants (4 males, aged 30.8±10.9 years, mean ± SD). Participants had no history of neurological impairment and were assessed as right hand dominant using the Edinburgh Handedness Inventory [24]. Written informed consent and the successful completion of a TMS safety screen [25] were obtained from each participant before running the experiments. All experimental protocols were approved by the Ethics Committee of the Université catholique de Louvain (Belgium).

### Manipulandum

The task consisted of grasping and lifting a 275-g manipulandum. This device comprised two 3D force-torque sensors (Mini 40 F/T transducers; ATI Industrial Automation, Garner, NC, USA), each one covered by a brass surface (40 mm diameter, 30 mm apart). The three orthogonal forces (F_x_, F_y_ and F_z_) were recorded by each sensor. Force signals were digitized on-line at 1 kHz (12-bit 6071E analogue-to-digital converted in a PXI chassis, National Instruments, Austin, TX, see [26]) and low-pass filtered at 15 Hz (fourth order, zero phase lag Butterworth filter) before any further processing. The grip force was calculated as the average of the normal forces (F_z_) applied by the thumb and the fingers on each transducer. The magnitude of the load force (LF) was computed as 
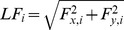
, where F_x,i_ and F_y,i_ are the horizontal and vertical components of the load force of transducer i (i = 1,2) respectively.

### Transcranial magnetic stimulation (TMS)

TMS was delivered through a figure-of-eight coil connected to a rapid model 200 stimulator (Magstim, Whitland, UK). Single pulse TMS was initially delivered over M1 to determine the optimal coil position to induce motor evoked potentials (MEPs) in the First Dorsal Interosseous (FDI). The coil was held over the contralateral hemisphere, tangentially to the skull with the handle pointing laterally and backwards at an angle of about 45°. Resting motor threshold (rMT), defined as the minimum intensity necessary to evoke MEPs of 50 µV peak-to-peak amplitudes in 5 out of 10 trials, was measured separately for right and left FDIs. No significant difference was found between the rMT determined for right and left FDI (42±7% of max stimulator output, mean ± SD; paired t-test, t_12_ = 0.034; p = .970).

### Stimulation sites

Because SMA is located directly anterior to the leg representation in M1 [27], [28], in order to target this area, we first determined the optimal coil position for evoking MEPs in the contralateral Tibialis Anterior muscle (TA) and we marked a two-centimetre point on the scalp anterior to this stimulation site [29]. This landmark was co-registered on individual anatomical magnetic resonance images for each participant by using a previously validated neuronavigation technique described in detail elsewhere [30], [26], [31], [32]. If necessary, the coil position was then slightly adjusted on the basis on the anatomical landmarks typically used to localize SMA namely, the most medial part of the superior frontal gyrus, dorsal and anterior to the precentral gyrus [33]. The coordinates of the stimulation point were then recorded and were close to the coordinates of SMA activation loci reported in the functional neuroimaging literature [16], [34], [13]. This neuronavigation procedure was performed for each volunteer before each experimental session and separately for each hemisphere.

**Figure 1:**
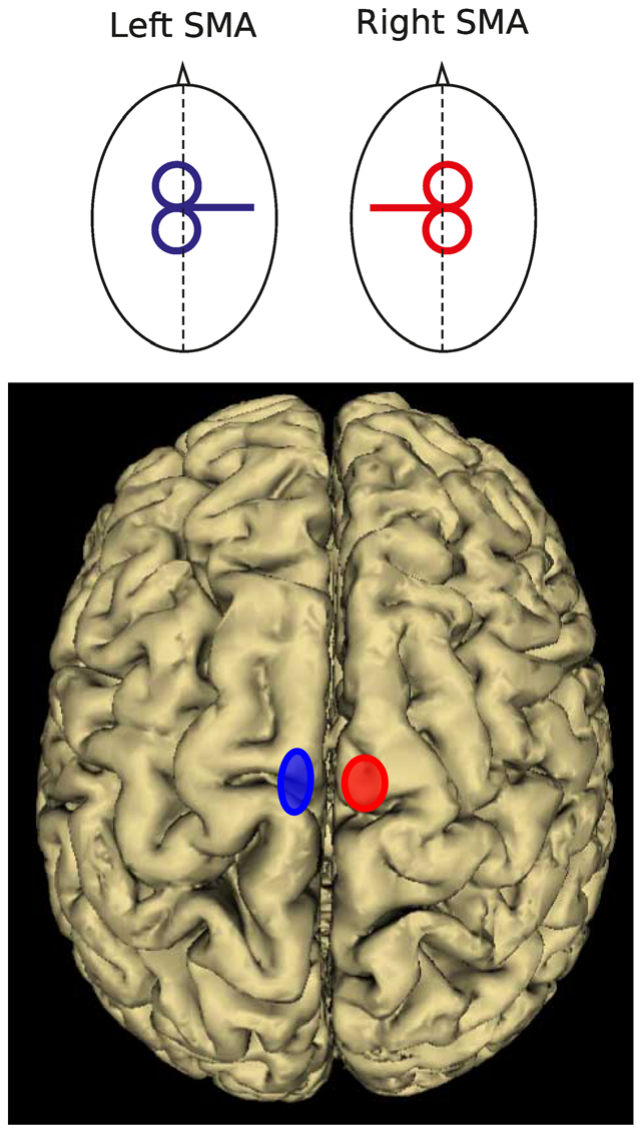
Coil position for optimal stimulation of left and right SMA (top) and mean location of the stimulation points over left (blue; −6.6, −6.4, 73.5; x, y, z) and right (red; 8.6, −6.9, 73.2; x, y, z) SMA following normalization into the MNI space (bottom). The centre of each ellipse is located over the mean MNI coordinates of each stimulation site. The area of the ellipses indicates the 95% confidence interval.

The coordinates of all stimulation sites were then normalized a posteriori into the Montreal Neurological Institute (MNI) system. For all volunteers participating in Experiments 1 and 2 (see below), the mean normalized MNI coordinates for left SMA were −6.6±2.4, −6.4±5.2, 73.5±5.8 mm (x, y, z, mean ± SD, n = 13) and 8.6±3.3, −6.9±4.4, 73.2±6.6 mm for right SMA, (x, y, z, mean ± SD, Figure 1).

### Experiment 1: Role of SMA in GF scaling

Eight participants volunteered for this experiment (7 males, aged 28±2.4 years, mean ± SD). Repetitive transcranial magnetic stimulation (rTMS, 10 Hz, 400 ms, 5 pulses) was applied either over the right or left SMA. As a control condition, rTMS was applied over the same areas with the coil in a sham position, perpendicular to the scalp. In order to target SMA, the coil was orientated medio-laterally with the handle pointing towards the unstimulated hemisphere (e.g. handle pointing towards the right hemisphere for left SMA stimulation, see Figure 1). This orientation induced a medio-laterally directed current, which has been shown to be optimal for stimulating the M1 leg area [35]. Repetitive TMS trains were separated by at least 12 s. Stimulation intensity was set at 120% of FDI rMT. Before each experiment we verified that, at this intensity and with the coil in this position, rTMS applied over SMA did not induce a twitch in either the ipsilateral or contralateral TA muscle. All electromyographic (EMG) recordings were made using silver/silver chloride surface electrodes positioned in a belly-tendon montage. Signals were amplified (Neurolog, Digitimer, Hertfordshire, UK) and sampled at 5 kHz in the bandwidth 20–1000 Hz (CED, Power1401, Cambridge, UK).

Before each experiment, subjects washed their hands with soap and water and dried them thoroughly. An auditory GO signal indicated trial onset and was followed, approximately 3 s later, by a second auditory signal marking the end of the trial. Repetitive TMS was delivered concurrently with the GO signal. Participants were instructed to grasp and lift the manipulandum using the minimum force necessary to prevent slips. The experiment consisted of 6 blocks of 12 trials with the right hand followed by 6 blocks of 12 trials with the left hand. The six blocks per hand were organized as follows: (i) 2 blocks with rTMS delivered over left SMA, (ii) 2 blocks with rTMS delivered over right SMA and (iii) 2 blocks, 1 over each SMA, performed with the coil in a sham position. Block order was counterbalanced across participants. Between each trial, participants were asked to adopt a rest posture with the hand resting on its ulnar edge, midway between pronation and supination, and the index finger and thumb positioned approximately 20 mm apart from the manipulandum grip surfaces.

### Experiment 2: Time course of left SMA contribution to GF scaling

Six subjects (5 males, aged 28±1.7 years, mean ± SD) participated in this experiment (two having participated in Experiment 1). The task and experimental procedure were the same as in Experiment 1 except that here we used paired-pulse TMS delivered using a bistim module (Magstim Company, Dyfed, UK) through a figure-of-eight coil. The coil position was determined using the same neuronavigation technique as described above. TMS intensity was set at 120% of rMT for the right FDI. Paired-pulse stimuli, separated by 5 ms, were delivered at seven equidistant delays after the GO signal during the movement preparation, namely 0, 50, 100, 150, 200, 250 and 300 ms; an eighth condition was a “no TMS” control condition. In a previous study, we used the same procedure successfully to pinpoint the time course of the anterior intraparietal area(AIP) contribution to grasping movements [36].

This experiment consisted of 8 blocks of 24 trials. Four blocks were performed with the right hand while paired-pulses were delivered over left, contralateral, SMA in the eight different delay conditions (7 timings + 1 no-TMS). The remaining four blocks were performed with the left hand with paired-pulse TMS delivered over right, contralateral, SMA. Three trials were performed for each TMS condition within each block and, thus, 12 trials per condition were available for analysis at the end of the experiment. The stimulation sites and hand were tested in a pseudo-random order across participants.

### Experiment 3: M1 control experiment

Finally, in order to determine whether the coil orientation adopted in Experiments 1 and 2 allowed us to target specifically the medial frontal region of only one hemisphere and rule out any significant spread of current to the opposite hemisphere, we applied the same rTMS protocol as in Experiment 1 (10 Hz, 400 ms, 5 pulses, 120% rMT of the right FDI) with the coil in a similar orientation but located over the leg representation of left M1. As already mentioned, 5 healthy subjects (4 males, aged 30.8±10.9 years, mean ± SD) participated in this control experiment. EMG signals were recorded simultaneously from the contralateral and ipsilateral TA muscles. Because of the difficulty to elicit reliable MEP in contralateral TA, rTMS was applied while participants performed a gentle contraction of both TA. At least ten EMG traces were recorded in each participant.

### Data analysis

Force signals were time-locked with the GO signal. GF and LF were computed together with their first derivatives (finite difference algorithm). GF and LF onsets were defined when the force exceeded the mean of the baseline value by 2 SD [26], [36]. We also measured peak of GF and mean GF during the static phase, defined as the interval between 500 and 1500 ms after object lift-off (reported by load force onset).

Temporal parameters of the grip-lift task were also measured: (1) reaction time (GO – T0; delay between the GO signal and first fingertip contact with the manipulandum); (2) duration of the preload phase (T0 – T1; delay between GF and LF onsets), (3) duration of the loading phase (T1 – T2; delay between LF onset and the moment LF equals the object's weight).

In addition, we calculated the largest coefficient of correlation between grip and load force rates and the time shift for which this condition was fulfilled (cross-correlation). These two values were computed for each individual trial and provided an estimate of the overall synergy of the grip-lift movement. Correlations quantified how well grip and load force profiles matched, which indicated the accuracy of anticipatory scaling of GF and time-shifts provided a measure of the asynchrony between the two forces. A positive time-shift indicates that GF leads LF, as it is usually reported in healthy humans [9].

In Experiment 3, EMG signals were rectified and integrated over 500 ms preceding the first rTMS pulse (baseline_EMG_) and for a period of 500 ms after this first rTMS pulse in both the ipsilateral (ipsilateral TA_EMG_) and contralateral TA muscles (contralateral TA_EMG_).

### Statistical analysis

There was no statistically significant difference between the movement parameters collected for the left and right SMA sham blocks in Experiment 1 (t-test; all t_7_>1.32; all p>.230). Therefore, we merged data in these two conditions and used them as control values. Statistical analyses were performed on all the aforementioned parameters using a 2-way repeated measures analysis of variance (ANOVA_RM_) with factors tms condition (left SMA, right SMA or controls) and hand (left or right hand). In Experiment 2, data were analysed using a 2-way ANOVA_RM_ with factors tms condition (left or right SMA) and tms timing (0, 50, 100, 150, 200, 250, 300, no TMS). When appropriate, post-hoc t-tests analyses were conducted. Data collected from Experiment 3 were analysed separately for left and right TA muscles. The EMG activity induced by rTMS was compared with baseline EMG using paired t-tests.

## Results

### Effect of left SMA virtual lesions on GF peak

**Figure 2:**
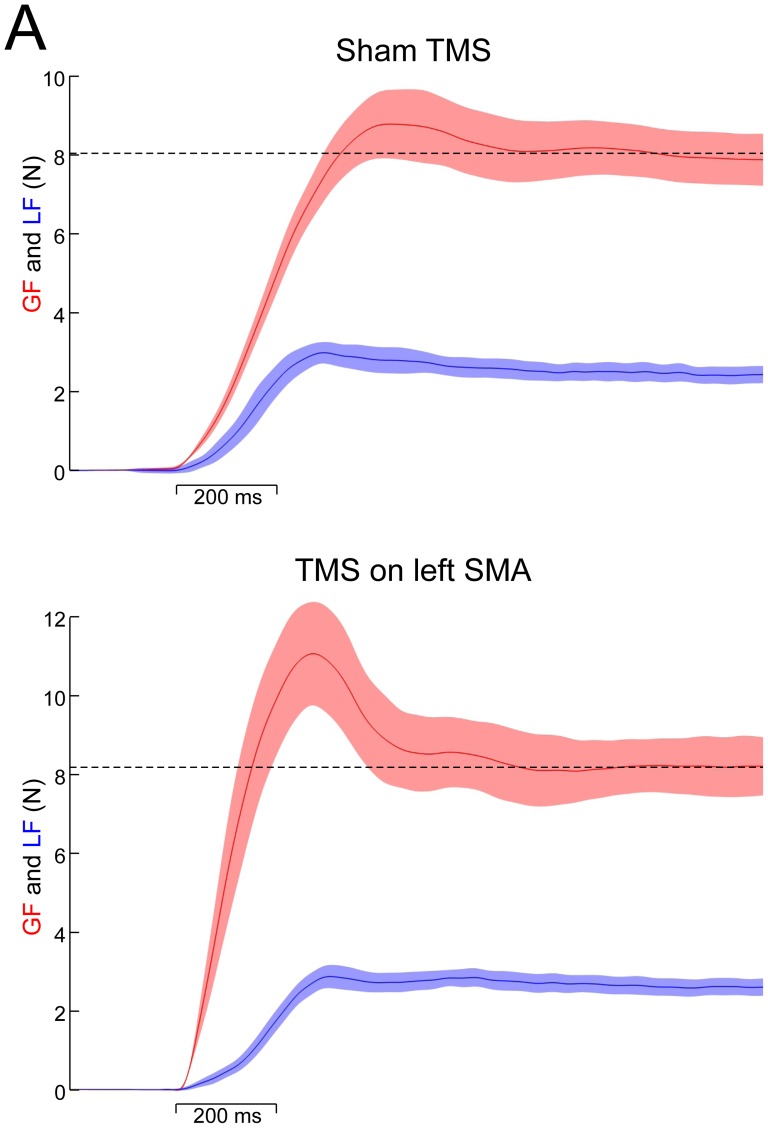
(A) Upper panel: mean grip (red) and load force traces (blue) over 12 trials under sham conditions in one representative participant. Lower panel: effect of a virtual lesion induced by TMS applied over left SMA on grip and load forces in the same participant. Dashed lines are positioned at the level of GF static. Error shade areas denote SD. (B) Effect of a virtual lesion induced in left SMA, right SMA and under the sham condition on peak GF. A significant increase in peak GF was evident following left SMA stimulation, regardless of the hand used. However, a further significant increase was present when left SMA stimulation was coupled with a right hand grasping movement. There was no significant increase in peak GF following right SMA stimulation. * p<.05.

When lifting the same object repetitively, subjects normally adopt stereotyped GF profiles. In particular, GF peaks just before the object is stabilized and then slowly converges to a settle point, which depends on the object weight and on other physical properties. Figure 2A illustrates GF and LF averaged over 12 trials in one representative participant in the control condition (sham, upper panel) and when TMS was applied over left SMA (lower panel). Both GF and LF increased in parallel in these two conditions. However, an overshoot in GF, as evidenced by a larger/higher/increased GF peak, was observed only when rTMS was applied over left SMA (see arrow) compared to sham condition and right rTMS stimulations.

The ANOVA_RM_ with tms condition (left SMA, right SMA or sham) and hand (left vs. right) showed a significant interaction for GF peak (F_1,28_ = 5.9, p = .002, Figure 2B). A post-hoc analysis revealed that, when the task was performed with the right hand, TMS applied over left SMA produced a 20% increase (about 2N) in GF peak when compared with right SMA TMS (t_7_ = 6.4, p<.001) and with sham (t_7_ = 8.7, p<.001) conditions. Similar results were obtained for grip-lift movements performed with the left hand, for which only left SMA TMS led to a significant GF peak increase when compared with right SMA (t_7_ = 5.4, p<.001) and sham (t_7_ = 4.7, p = .009) conditions. Interestingly, the effect of left SMA TMS on GF peak was significantly larger for the right, contralateral hand, than for the left hand (t_7_ = 5.3, p<.001, Figure 2B). So, TMS applied over left, but not right, SMA altered GF scaling, and this effect was slightly larger when participants used their right, contralateral, hand to perform the task.

Accordingly, peak GF rate, which reflects the speed at which muscle fibres are recruited, were also altered by TMS applied over the left SMA. Indeed the ANOVA_RM_ revealed a main effect of tms condition on peak GF rate (F_1,28_ = 12.4, p<.001), which increased significantly when rTMS was applied over left SMA TMS when compared with right SMA (t_7_ = 4.3, p = .007) and sham (t_7_ = 5.7, p = .003) conditions. No tms condition x hand interaction was found which indicates that this effect was identical for both hands.

Finally, it is worth mentioning that a main effect of tms condition was also found for the maximal coefficient of correlation computed between first derivatives of GF and LF (F_1,28_ = 5.2, p = .006) whereas time shifts remained unaffected (see Table 1). Indeed, the maximal correlation was significantly reduced following left SMA stimulation when compared with both right SMA (t_7_ = 4.9, p = .012) and sham (t_7_ = 3.7, p = .026) conditions and this effect held regardless of the hand involved in the task (ANOVA_RM_, no hand nor tms condition x hand effects, all F<1). This lower coefficient of correlation is likely due to the change in GF rate following left SMA TMS (see above), LF rate remaining unaffected by TMS.

**Table 1:** Mean (SD) values of movement parameters for each stimulation site (Left vs. Right SMA) and hand condition (LH, Left Hand; RH, Right Hand) in Experiment 1.

Variable	Control	Left SMA		Right SMA	
		*LH*	*RH*	*LH*	*RH*
Peak GF (N)	9.2 (1.7)	11.3 (2.1)*	12.8 (1.9)*	9.2 (1.2)	9.5 (1.9)
Peak LF (N)	3.8 (.6)	3.9 (.6)	3.9 (.6)	3.9 (.6)	3.9 (.6)
GF static (N)	8.4 (1.2)	9.1 (1.4)	8.9 (1.5)	8.7 (1.1)	8.8 (1.2)
Peak GF rate (N/s)	66.6 (14.6)	80.1 (13.2)*	83.9 (15.5)*	76.9 (18.9)	75.4 (18.4)
Peak LF rate (N/s)	31 (10.3)	33.4 (8.3)	30.7 (9.2)	28.4 (10.1)	33.5 (9.8)
Correlation coefficient	0.85(.06)	0.82 (.07)*	0.83 (.07)*	0.84 (.07)	0.83 (.07)
Time-shift (ms)	14.6 (18)	12.3 (20.1)	15.1 (17.5)	11.8 (19.7)	14.7 (22.6)
Preload duration (ms)	37.5 (24.2)	38.9 (21.5)	27.4 (16.4)*	25.6 (23.2)*	37.4 (22.0)
Loading phase duration (ms)	179 (19.6)	191 (19.2)	185 (22.1)	187 (19.4)	183 (22.0)
Reaction time (ms)	353.5 (39.5)	374 (21.9)	385 (58.5)	381 (30.1)	338 (41.2)

Control values correspond to average between left and right hands in the Sham condition, see Methods. Asterisks (*) denote statistical difference with control values at p<0.05.

As shown in Table 1, other grip-lift parameters (reaction time, LF peaks, GF static, time shift, and load phase duration) were not significantly different from control values (t-test; all t_7_>2; all p>.078), indicating that these variables were not influenced by rTMS applied over SMA. Importantly, GF static was adjusted suitably according to the object's weight. The dynamics of object loading, as indicated by peak LF rates were also similar (t = 2.2, p = 0.067). In addition both loading phase durations and reaction times were identical across conditions (t-test; all t_7_>1.6; all p>.145).

### Time course of left SMA contribution to GF scaling

**Figure 3:**
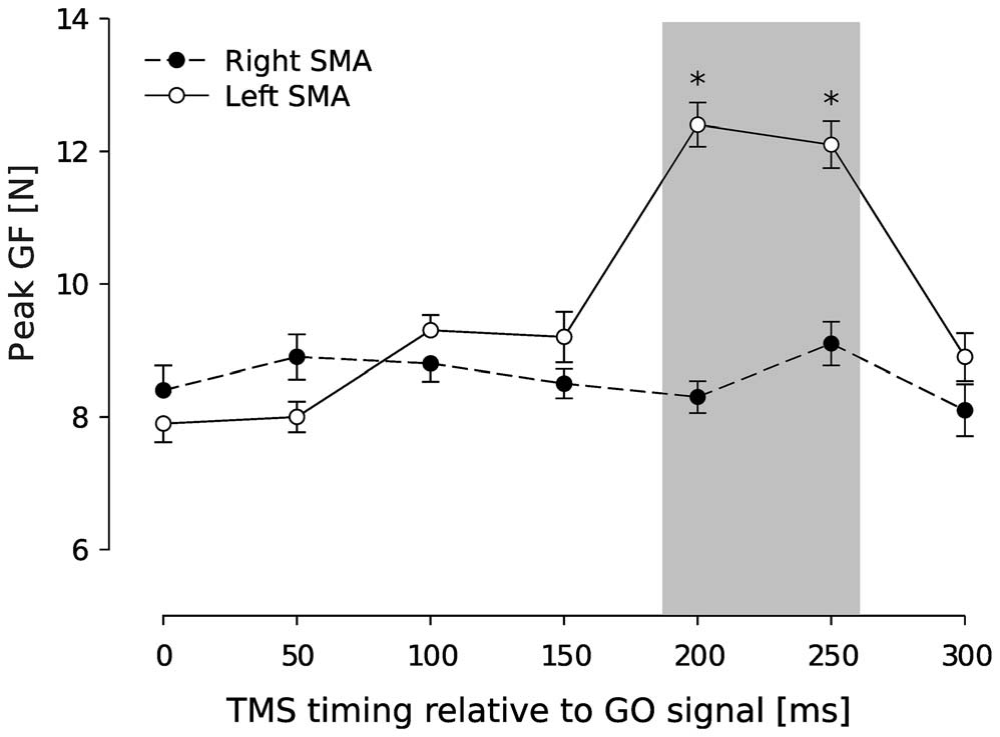
Time course of the SMA contribution to GF scaling. Circles represent the mean (±SE) of GF peaks obtained at each epoch when TMS was applied on right SMA (closed circles) and left SMA (open circles). Peaks of GF increased significantly when paired-pulse TMS was applied over left SMA at 200 and 250 ms after the GO signal. No increase was seen in peak GF following paired-pulse TMS applied over right SMA (*p<0.05).

In Experiment 2, paired-pulse TMS was applied either over left or right SMA while participants performed the grip-lift task with the contralateral hand. This experiment allowed us to investigate the time course of left SMA contribution to GF scaling during movement preparation since, based on results gathered in Experiment 1, we expected an effect on GF scaling only in left SMA condition. Figure 3 illustrates GF peaks gathered for each delay of TMS application over the right SMA (closed circles, left hand) and over the left SMA (open circles, right hand). An ANOVA_RM_ unveiled a significant tms condition x timing interaction (ANOVA_RM_, F_6,84_ = 2.9, p = .007) on GF peak and post-hoc revealed that TMS led to a significant increase in GF peak only when delivered over left SMA between 200 and 250 ms after the GO signal (t_5_ = 3.8, p = .005; Fig. 3). Taking into account the mean reaction times in this task (385±58.5 ms, mean ± SD), this indicates that left SMA influenced GF scaling during movement preparation, approximately between 180 and 130 ms before initial finger contact was made with the object.

### Role of SMA in controlling preload phase during movement preparation

**Figure 4:**
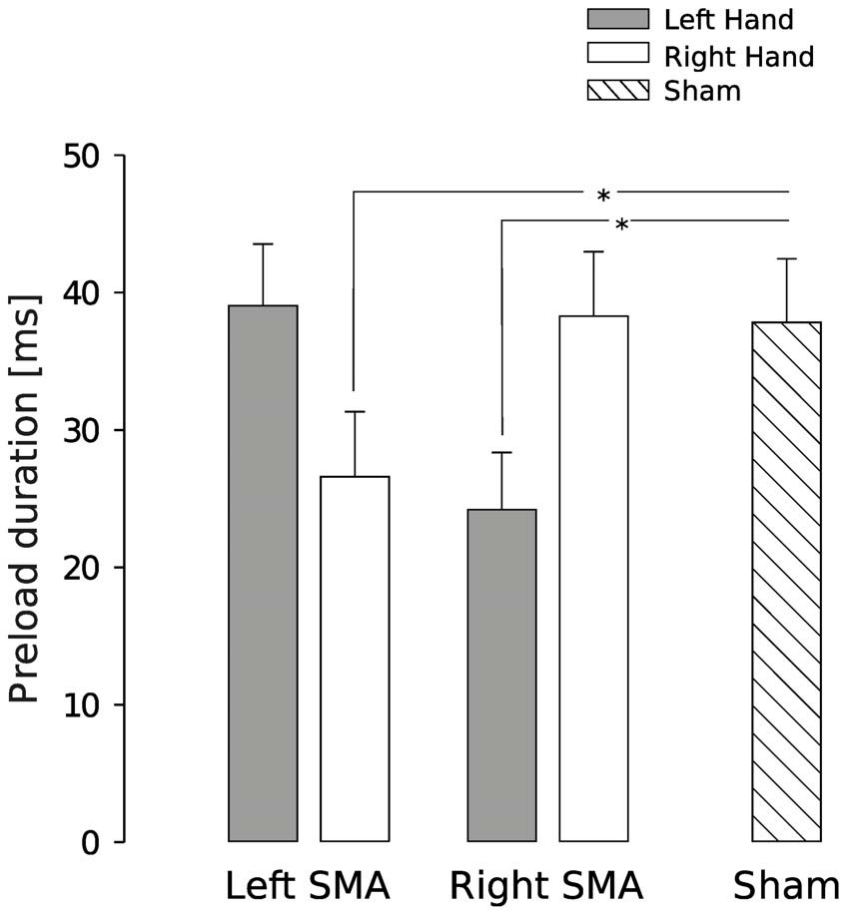
Effect of a virtual lesion induced in left SMA, right SMA and under the sham condition on preload duration. Both left SMA and right SMA stimulations produced a significant decrease in preload duration compared to sham, but only when the contralateral hand was used to complete the task. * p<.05.

The preload phase, i.e. the delay between object-finger(s) contact and the first increase in LF, is an important variable to consider in a grip-lift task because, during this short period of time, physical properties of the object are encoded by mechanoreceptors, prior to loading the object. Figure 4 reports mean preload phase durations when TMS was applied over the left or right SMA or in the sham condition, for the left (closed bars) or right hand (open bars). The ANOVA_RM_ showed a significant tms condition x hand interaction (F_1,28_ = 7.9, p<.001) and post-hoc analysis indicated that the preload phase duration significantly decreased by about 30% in left (t_7_ = 4.3, p = .007) and right SMA conditions (t_7_ = 3.7, p = .012) when compared with the sham condition. However, this effect held only when the hand contralateral to the TMS stimulation was involved in the grip-lift task (left SMA TMS: right hand > left hand, t_7_ = 3.3, p = .027; right SMA TMS: left hand > right hand, t_7_ = 3.8, p = .018).

### Lack of current spread to the opposite hemisphere

**Figure 5:**
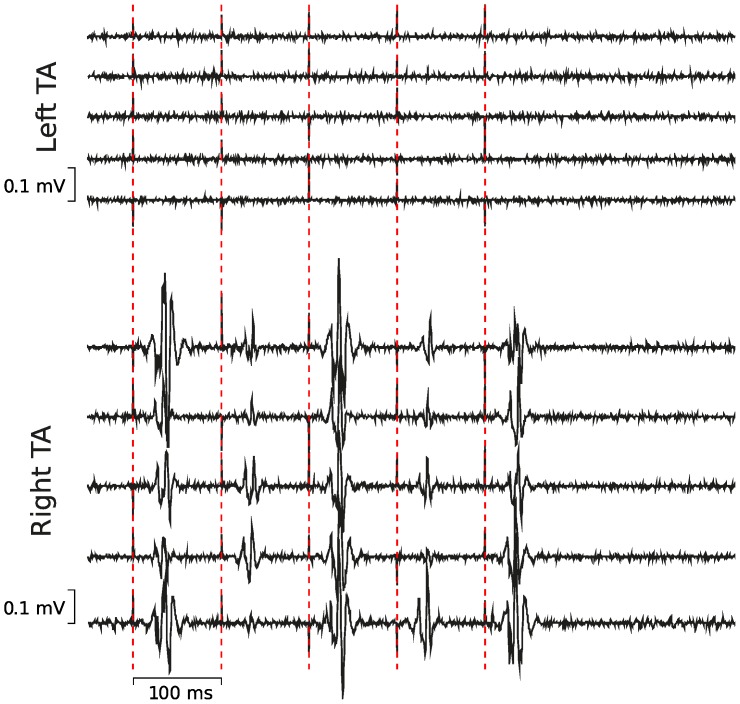
Control experiment (Experiment 3) was designed to rule out any spread of rTMS between hemispheres. While delivering rTMS over the left tibialis anterior (TA) M1 representation, we recorded MEPs in the right TA (bottom five traces) but not in the left TA (upper five traces). Vertical cursors are positioned at TMS pulses.

Because left and right SMA are next to each other, in order to rule out that rTMS applied over one SMA induced a spread of current into the contralateral SMA, we ran a control experiment (Experiment 3, Fig. 5) in which rTMS (same configuration as in Experiment 1) was delivered over the TA muscle representation in left M1. Since the TA motor representation has the same location on the edge of the interhemispheric sulcus as SMA [28], we reasoned that if a significant current spread between both SMAs occurred, in this control experiment, rTMS should also elicit MEPs in the ipsilateral TA.

We failed to record any change in EMG activity in ipsilateral TA following rTMS applied over the left leg representation of M1, when participants were asked to maintain a small voluntary contraction. Indeed, no MEPs were elicited in the left, ipsilateral TA, and the EMG activity recorded during rTMS (see Methods) was undistinguishable from that of the baseline (t_4_ = 1.25, p>.05, Fig. 5 upper five traces). In contrast, as expected, in the right, contralateral TA, rTMS induced MEPs in a systematic manner and the area under the rectified EMG was significantly larger during rTMS than in the baseline (t_4_ = 10.3, p = .001, Fig. 5, lower five traces).

## Discussion

The present study reveals the critical contribution of SMA in a grip-lift task. First, we found that rTMS applied over the left, but not right, SMA led to an increase in GF peaks in both hands. These effects were present only when TMS was applied within a given time window, namely 180–130 ms, before fingers contacted the object. Second, we showed that both right and left SMA are involved in the preparation of movements performed with the contralateral hand, as evidenced by a decrease in preload phase duration. These findings suggest that the functional roles played by the left and right SMA can be dissociated.

Several studies have already suggested that SMA is involved in GF scaling. In monkeys, Smith et al. showed that a lesion of SMA led to an excessive GF and GF rate, accompanied by a “forced grasping”, defined as a difficulty to release the object [37]. However, further attempts to correlate neuronal activity in monkey SMA and GF, or GF rate, remained unsuccessful [38], [39], suggesting that SMA is not responsible for controlling low-level processes but instead in higher-processes underlying GF scaling, such as adaptation. In humans, many functional imaging studies have shown that SMA is activated in tasks involving precision grasping [13], [14], [15], [16]. However, despite the findings that SMA activation is correlated with the degree of GF precision needed during grasping [15] and with the force exerted during a key-press task [40], results from imaging studies remain inconclusive about the causal contribution of SMA to GF scaling.

The present study provides the first experimental evidence that left SMA is causally involved in GF scaling during movement preparation. However this does not tell us what are the neural processes taking place in left SMA and in which operation on internal models this area might be involved. Motor control relies on a mixture of predictive and feedback mechanisms which allow us to perform multiple actions in a dynamic environment; this view has been successfully conceptualized through the theoretical framework of internal models [41]. For example, when lifting objects, the sensorimotor system predicts the sensory events associated with object lift-off, e.g. tactile afferents. If a mismatch between predicted and actual sensory information is detected, the system can launch appropriate, task-protective corrective actions and can also update the estimates of parameters characterizing object properties to improve future actions. Initially, prior knowledge about the task context helps choosing the most likely model that will be reused until an error is detected [42], [6]. So far, only the neural correlates of internal model storing have been assigned to the ipsilateral cerebellum [5], [17], [20], [16], [19] but the brain structures responsible for selecting and loading the correct internal models are still unknown.

The present study shows that left SMA virtual lesions led to a systematic increase in GF force peak regardless of the hand used to perform the grip-lift task. Because there is no evidence in the literature that a SMA lesion alters force production per se, it is sensible to assume that the inappropriately high GF peaks - for similar load forces - we observed after a left SMA virtual lesion reflect a failure to predict the required GF given estimates of object dynamics. In other words, this suggests that a left SMA virtual lesion prevents the loading of the appropriate internal model. Results from Experiment 2 further indicated that GF scaling is only affected between 180 and 130 ms before object contact following left SMA TMS. However, we cannot rule out that left SMA TMS might have simply altered GF gain. Indeed, GF depends on object properties and, for instance, gains are higher while lifting heavy objects as compared to lighter ones. Whether TMS applied over left SMA would yield a consistent increment of GF in light and heavy objects or would lead to an alteration of gains unrelated to the object dynamics remains to be investigated. New experiments should be performed with objects with different properties (e.g. weight and friction) to show that TMS applied over left SMA does not alter GF gains as a function of these properties.

By considering both the bilateral effects of left SMA TMS on GF peak and the contralateral effects of left and right SMA TMS on the preload phase duration, one can attempt to disentangle the roles of the two SMA. When TMS was applied over left SMA, GF peaks significantly increased in both hands although the preload phase duration was shorter only for the right, contralateral, hand. In contrast, GF peaks remained unaffected when rTMS was applied over the right SMA despite the fact the preload phase duration decreased when the task was performed with the left, contralateral, hand. This suggests that the left SMA is involved in GF coding independently of the effector whereas preload duration is controlled by each hemisphere in an effector-dependent way. Alternatively, it could be argued that left SMA rTMS could have prevented the update of parameters of the internal model. However, participants performed the task in different intermixed conditions, including sham, which were used to refine internal object representation. The questions remain open as to why GF peaks were systematically larger, and not smaller, if the correct internal model was loaded and why a dramatic decrease in preload phase duration did not affect task performance when TMS was applied over the right SMA. The present pattern of results reflects a complex interplay between high-level processes that code object representation in an effector-independent way, in the left SMA, and low-level processes that translate the representation into motor commands for the contralateral effector, in both the left and right SMA. A prediction of these interpretations is that adaptation should be impaired if participants learn to manipulate more complex object dynamics. This should provide another window into uncovering what feature is exactly encoded during the preload phase.

Interestingly, anatomical studies in monkeys have shown that most motor, premotor and parietal areas involved in controlling grasping movements are interconnected with SMA [43], [33], [27], [44], [45], [46]. SMA is, indeed, heavily interconnected with PMv, PMd and, to a lesser extent, with M1 [47], [45], [48]. Surprisingly, amongst these areas belonging to the grasping circuit [49], [50], only AIP is not connected to SMA [51] although we found in a previous TMS study that AIP also plays a causal role in GF scaling [36]; this suggests that SMA and AIP might control GF independently, depending on the experimental conditions. In addition, Akkal et al. [52] have shown that SMA is also a target of the cerebellum and basal ganglia, indicating that this area is part of the cortico-cerebellar and cortico-basal ganglia loops involved in controlling grasping movements. In particular, basal ganglia have been shown to be involved in the control of GF [53]. Although it is still difficult to speculate on the respective contribution of these different pathways to the control of the multiple aspects of grasping movements, it suggests that there may exist several parallel and independent channels to control GF, possibly under different circumstances.

The present results also point out towards a dominant contribution of the left SMA in GF scaling, in agreement with our previous finding that only left AIP lesion led to a deficit in GF scaling in a similar task [36]. However, this conclusion about a left hemispheric dominance for the representation of internal models is only valid if we can rule out any spread of current towards the right SMA while stimulating the left SMA. Several arguments support the specificity of our effects: (1) rTMS applied over right SMA failed to produce change in GF scaling, an effect remarkably consistent across participants and experiments and thus incompatible with a spread of current from the right to left hemispheres, and therefore the opposite must be true too; (2) rTMS applied over the TA representation in M1 also suggested that our stimulation procedure did not produce a substantial crosstalk into the opposite hemisphere.

The left hemisphere dominance for movements is usually regarded as specific to high-level cognitive functions, such as tool use [54]. Indeed, the involvement of left parietal and premotor cortex in apraxia indicates that the left hemisphere benefits from higher abilities than the right hemisphere in the representation of actions [55]. This representational role has been linked by some authors to the ability of the left hemisphere for storing declarative knowledge about objects through language [56], [57]. However, the present results challenge the idea that the left hemispheric dominance in object-related actions is confined to a representational level. Indeed, the dominance of the left SMA and AIP in GF scaling when manipulating simple objects, shows that the left hemisphere dominance is also reflected in elementary aspects of movements [58], [36]. Interestingly, a thorough investigation of movement kinematics has shown that apraxic patients have some deficits in grasping movements [59]. Together with the present results, this indicates that left hemisphere lesions may impair lower-level motor processes, which might exacerbate higher-level deficits usually observed in patients with limb apraxia.
